# Editorial

**Published:** 1860-09

**Authors:** 


### Editorial.

### AMERICAN DENTAL CONVENTION.

This body met at Saratoga on the 7th of August, and continued its sessions till Friday, the 10th, at one o'clock, P. M. It was conceded by all whom we heard give an expression of opinion, that it was the best meeting of the kind that has ever been held.

There was not much in the way of deep, extensive research; but few essays were read; but there was much elicited in the way of short discussions that was new, interesting and valuable; there were more useful points elicited than at any former meeting. There was a genial freedom of expression, which was refreshing to see. The utmost harmony prevailed, and though there were differences of opinion, they did not engender unpleasant controversy. It would be pleasant here to bring together in brief, some of the valuable ideas they brought forth; but a few samples must suffice; we give a few as they occur to our mind, not that they are better than many others, that we do not give; and first,

Dr. *Clark's* method of procuring metallic model and counter model without the use of sand or plaster, farther than the impression.

Dr. *Rogers'* method of applying arsenic through perforated wax to the pulps of the teeth.

Dr. *Westcott's* methods of applying arsenic, and the principle upon which he does it.

Dr. *Clay's* method of continuous gum work.

Dr. Allen's method of inserting temporary work.

Dr. Roberts' method of mounting partial sets of teeth.

Dr. Wetherbee's method of inserting full sets of teeth.

Ideas upon correcting irregularities.

Several new and improved implements and appliances were exhibited, prominent among which were improved vulcanizers, vulcanite emery wheels, etc.

The many attractions of Saratoga operated somewhat against the constant attendance of many of the members; but that is to be charged to the attractions, rather than to the members.

The adjournment was made to New Haven, Ct., where we hope to see our Convention held free from outside influences, as this was one strong argument used by the friends of that location, while those opposed to that location contended that the members would not go without some outside attraction. Well, we will see. T.

### AMERICAN DENTAL ASSOCIATION.

The delegates from the various existing dental associations met in Washington City July 31st, 1860, for the purpose of forming a national association. The local associations were pretty generally and fully represented. They met and operated togother in effecting the organization, with the utmost harmony. Not very much was done in the way of discussing practical points, from the fact that the time was mostly occupied in effecting the organization. This was done deliberately and with great care.

There were three or four papers read, which will be published. We do not suppose it will be the object of this association to enter very largely into the oral or extemporaneous discussion of subjects, but through the appropriate committees endeavor to elicit thoroughly digested and well written essays upon the various principles involved in the art and science of the profession.

In this respect it will differ materially from the American Dental Convention, at least in its operations thus far.

In the National Association, committees are appointed, whose duty it is to investigate the various subjects pertaining to dental science, and embody the results in well written, comprehensive essays, that may in some sort be referred to as the extent of attainments in the particulars treated of. These committees have a whole year in which to prepare their reports, and if they are industrious and energetic, which no doubt they will be, such progress will be made in dental science and art, as has not yet been dreamed of. We expect that many subjects will be brought forth, and made to render tribute, that have not heretofore entered into dental discussions, and those that have will be more thoroughly examined and considered. T.

### MOUNTING TEETH WITH DOUBLE PLATES.

We notice that Dr. Meriman has the patent for constructing sets of teeth with double plates; the teeth are soldered to a narrow base plate. This narrow plate, after the teeth are attached, is riveted to the main plate, and in this way heating the plate is avoided, and consequently the springing that so often occurs in this process.

It is said by those who have used it, that a piece of work is quite as easily and expeditiously put up as by the common method; the work is easier finished than the ordinary work. We have seen the same principle applied in the construction of continuous gum work, the teeth and gum put upon a narrow base plate of platinum, and this riveted to a main plate of gold.

There is recently introduced to the notice of the profession another method of attaching teeth to gold plates, without heating the plates to warp them; this is by an attachment of velcanized rubber; of the efficiency of this we do not know personally, but much is claimed for it.

Of the patents connected with these methods we now have nothing to say; on the subject of dental patents, we intend some time to express ourself. T.

### SERUM IN PULP CAVITIES.

In conversation recently with Dr. Allport, on the probability of the passage of serum through dentine, he remarked, that he entertained scarcely a doubt that the two apparently different opinions entertained upon the subject were perfectly reconcilable, and that there was no real ground for the controversy that has existed upon the subject.

He remarked that from various experiments which he made, he does not entertain a doubt that the dentine of persons prior to middle age, and especially that of young persons, will afford a ready passage for fluid through it into pulp cavities and canals, and especially if these are sealed up so as to form a vacuum. But that, upon the other hand, he is as fully of the opinion that the dentine may be easily prepared, so as to preclude the passage of any fluid through it. This may be done by the application of creosote or any similar oil, applied to the dentine, which it absorbs, till it becomes saturated, and this prevents the passage of any other fluid. The treatment to which the fangs are ordinarily subjected, brings about this very desirable condition, when the pulp cavities are not to be filled. We do not remember to have seen this idea advanced before ; it is an important feature, and is applicable as well in cases where the fangs and pulp cavities are filled, as where they are not. Creosote is a very general application in the treatment of fangs, preparatory to filling or closing up. T.

A TREATISE

*On the medical history and treatmant of diseases of the teeth and the adjacent structures.* By B. W. Richardson, A. M., M. D.

This is a work recently issued by H. Bailliere, of London. It consists of the very able lectures delivered by Dr. Richardson before the members of the College of Dentists of England, during the session of 1858 and '59. It is a valuable acquisition to dental literature. In it many of the points of dental science are discussed and elaborated with great ability. A depth of thought and research are here exhibited, that is too seldom found in our dental literature. The work fully sustains the high reputation of the author. It is a work that every one making any pretensions to professional attainments, should have at hand. It may be obtained at the Dental Depots, and we presume at bookstores generally. T.

*Ventilation of Rooms at Night.*—An extraordinary fallacy is the dread of night air. What air can we breathe at night but night air ? The choice is between pure night air from without and foul night air from within. Most people prefer the latter.

An unaccountable choice. What will they say if it is proved to be true, that fully one-half of all the disease we suffer from, is occasioned by people sleeping with their windows shut ? An open window most nights in the year can never hurt any one. In great cities night air is often the best and purest air to be had in the twenty-four hours. I could better understand in town shutting the windows during the day than during the night, for the sake of the sick. The absence of smoke, the quiet, all tend to making night the best time for airing patients. One of our highest medical authorities on consumption and climate has told me that the air of London is never so good as after ten o'clock at night.—*Florence Nightingale.*

